# Eliminating the Deadwood: A Machine Learning Model for CCS Knowledge-Based Conformational Focusing for Lipids

**DOI:** 10.1021/acs.jcim.4c01051

**Published:** 2024-10-08

**Authors:** Mithony Keng, Kenneth M Merz

**Affiliations:** † Department of Chemistry, Michigan State University, East Lansing, Michigan 48824, United States; ‡ Department of Biochemistry and Molecular Biology, Michigan State University, East Lansing, Michigan 48824, United States

## Abstract

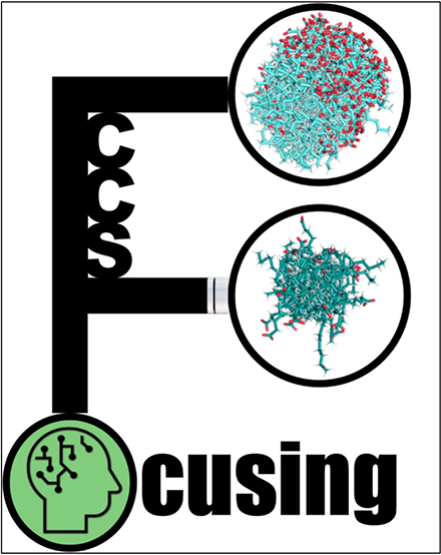

Accurate elucidation of gas-phase chemical structures using collision cross section (CCS) values obtained from ion-mobility mass spectrometry benefits from a synergism between experimental and *in silico* results. We have shown in recent work that for a molecule of modest size with a proscribed conformational space we can successfully capture a conformation(s) that can match experimental CCS values. However, for flexible systems such as fatty acids that have many rotatable bonds and multiple intramolecular London dispersion interactions, it becomes necessary to sample a much greater conformational space. Sampling more conformers, however, accrues significant computational cost downstream in optimization steps involving quantum mechanics. To reduce this computational expense for lipids, we have developed a novel machine learning (ML) model to facilitate conformer filtering according to the estimated gas-phase CCS values. Herein we report that the implementation of our CCS knowledge-based approach for conformational sampling resulted in improved structure prediction agreement with experiment by achieving favorable average CCS prediction errors of ∼2% for lipid systems in both the validation set and the test set. Moreover, most of the gas-phase candidate conformations obtained by using CCS focusing achieved lower energy-minimum geometries than the candidate conformations without focusing. Altogether, the implementation of this ML model into our modeling workflow has proven to be beneficial for both the quality of the results and the turnaround time. Finally, while our approach is limited to lipids, it can be readily extended to other molecules of interest.

## Introduction

Many *in silico* modeling experiments require generating an ensemble of conformers for a particular molecular system in an attempt to represent as much of the conformational space as possible to capture insights into the equilibrium structure and its associated thermodynamic properties. For the popular knowledge-based conformational generator that relies on force field implementation, the software is typically trained on data sets consisting of condensed-phase structures that have been retrieved from either the Protein Data Bank (PDB) or the Cambridge structural database (CSD).^1,2^ In practice, for the most part, parametrization using condensed-phase data allows for reasonably good agreement between the predicted chemical structures and their counterpart solution-phase experimental results. Despite results from studies^3,4^ showing that the current knowledge-based generators (*e.g*., ConfGen and OMEGA) transfer well to covering relevant gas-phase conformational space for small and midsize molecules, these favorable outcomes are, from our experience, likely an effect of sampling a large ensemble of conformers, low molecular flexibility, and/or subsequent treatment with high-level quantum mechanical (QM) optimization including dispersion interactions.

It should be noted that potential functions (*e.g*., OPLS, MMFF94) used in force field construction for conformation generators are formulated from classical mechanics.^5^ These methods, however, are not always good at finding true local minima unlike high-level QM methods (*e.g*., DFT, MP2, CCSD).^6^ In the solution, intramolecular dispersion interactions are attenuated because intermolecular interactions can dominate, especially in biologically relevant polar solvents where the formation of dipole–dipole, ion-dipole, ion–ion, and hydrogen bonding interactions is much more energetically favorable. However, in vacuum long-range electrostatic and dispersion interactions within the molecule itself (*i.e*., intramolecular dispersion) play a significant role in the preferred low-energy conformation. With small or rigid molecules, these intramolecular interactions play only a limited role in defining the topological diversity within a conformational space. On the other hand, when we consider a biomolecule like a fatty acid, which are flexible and largely nonpolar, the long-range dispersion interaction can lead to a gas-phase equilibrium conformation that may either be grossly underrepresented in an ensemble or completely missed using current conformation generators.

Additionally, a major bottleneck in high-quality modeling is the QM geometry optimization step, even when using an efficient computational chemistry theory like density functional theory (DFT) with a minimal basis set, it can still take on average several days for the processing of many sizable biomolecules. Therefore, it would be a best practice approach for a modeling workflow not only to reduce the number of irrelevant or “deadwood” conformers within an ensemble but, most importantly, to ensure that the ensemble contains enough relevant conformers. To the best of our knowledge, no method exists in the conformation generation regime that explicitly uses a gas phase chemical descriptor to directly dictate the conformation sampling process. Here, we introduce a novel machine learning (ML) model that executes a collision cross section (CCS) knowledge-based approach to focus the conformational space generated by most, if not all, currently available conformation generators. CCS or rotationally averaged surface area is an ion-mobility mass spectrometry (IM-MS) instrument generated molecular shape descriptor of gas phase structure and ion-neutral interaction.^7−9^ As we have alluded to earlier, the gas phase structures of fatty acids, along with other lipid subclasses, pose a formidable challenge to accurately resolve and thus are good candidate systems to challenge our model.

We want to emphasize that our CCS focusing (CCSF) engine is not a conformation generator *per se* but a conformation generation postprocessing filter that is built to complement most currently available generators. The model “batch-predicts”, with high throughput, the CCS values for an ensemble of conformers and from there retains only conformers with CCS values that are within a user-defined CCS threshold (*e.g.,* experimental CCS). At the earliest stages, the predicted CCS results allow a user the opportunity to amend or add additional features to a workflow to better improve the outcome of a structure prediction project. For example, a modeler can responsively increase the number of conformers per ensemble, use an alternative force field, or use a different conformation generator altogether. For handling lipids, we have opted to saturate the conformational space with as many conformers as needed since changing the force field and/or the generator would likely make a negligible difference on conformational space coverage.

In this work, we first executed a standard workflow to predict the gas phase structures of an inventory of test lipid species. The resulting conformations for each lipid are then scored for candidacy according to their DFT level relative energies. Next, the quality of the predicted candidate conformers is assessed by comparing their *in silico* derived CCS values against the experimental reference CCS. All CCS values used in this work exclusively involve nitrogen (N_2_) as the neutral collision buffer gas in the IM-MS experiments. We then proceeded to use the features and CCS label obtained from this initial workflow to train and build our CCS conformation focusing ML model. The completed model is then implemented in an augmented workflow (the standard workflow with the knowledge-based ML model incorporated), where it is validated for performance and generalizability.

## Computational Methods

### Charge Modeling and Conformation Generation

**Figure 1:**
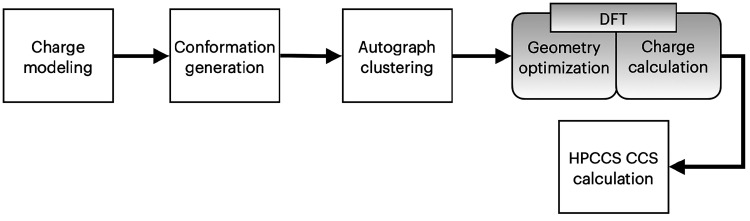
A schematic of the standard workflow for gas-phase structure prediction and source of data set.

Figure 1 shows the essential steps of our standard workflow for obtaining the gas phase lipid candidate conformations and their corresponding computed CCS values. It should be noted that this standard workflow is a modified version of our POMICS^10^ workflow. For the charge modeling step, all potentially proton donating or acidic sites, mainly the carboxylic acid group, were deprotonated to yield negative mode charge models, [M-H]^−^. For this task, Maestro^11^ was used to manually edit and remove the appropriate hydrogen atom (*i.e*., acidic hydrogen) one model at a time to give a unique anion model for each lipid ion system. Next, each lipid charge model was set to generate a standard ensemble of 1000 conformers using the conformation generator ConfGen.^12^ To eliminate structure redundancy within each ensemble, the software Autograph^13^ was used to cluster the conformers into representative conformer centers. With Autograph, the number of conformers was reduced, on average, from 1000 to <50, which made the workload more practical for the QM step.

### Conformer Geometry Optimization and Partial Charge Calculation

The QM calculations for the ensembles were carried out using the Gaussian16 software.^14^ The QM geometry optimization of the anionic conformer centers was processed at the DFT B3LYP/6-31+G(d,p) with D3BJ^15,16^ (Grimme empirical dispersion with the Becke-Johnson damping function) level of theory. Only the geometries that successfully converged numerically during the optimization step moved forward to the partial charge calculation step. To be consistent, the subsequent DFT charge calculations on the optimized geometries, employing the Mulliken^17^ charge scheme, were also done at the D3BJ-B3LYP/6-31+G(d,p) level of theory. The atomic partial charge values are required for subsequent *in silico* CCS calculation. The CCS values were computed using the open-source software HPCCS,^18^ which performs the calculation using the trajectory method.

### Candidate Conformer Determination

The selection of the gas phase candidate conformation for each charge model was based on scoring the relative energy of the DFT optimized ensemble with the lowest energy conformer being the selected candidate. In addition, the accuracy of our structure prediction was determined by comparing the Boltzmann-weighted (averaged) computed CCS to the experimental reference CCS.^19−26^ To account for uncertainties originating from IM-MS instrument calibration error, experiment settings, instrumentation, and the use of the Mason-Schamp equation to calculate CCS, we have used a 3% computed CCS error threshold.^27,28^ Thus, a lipid system with a predicted equilibrium structure that resulted in a CCS disagreement between experiment and prediction of >3% was considered for the additional ML augmented workflow (see Figure S1 in Supporting Information) for validating our ML model.

### ML Training Data Preparation

For the training data selection, only the pre-QM raw conformers from the test ensembles with successfully predicted CCS values obtained from the standard workflow were used to train the ML model. We deliberately tethered the raw conformers to the predicted CCS label, since requiring a QM optimized structure for training would make the model counterproductive and inimical to the justification of having the augmented workflow. In total, the data set constructed at the conclusion of this work contained ∼1200 instances (structural conformers) from 34 unique lipid anion, [M-H]^−^, species. A custom Python script was deployed on a Google Colab^29^ notebook to execute feature extraction and engineering from the input XYZ files. To ensure high throughput during the model training and prediction tasks with large ensembles, we constrain our features to five relevant molecular level descriptors: mass-to-charge ratio (mz), farthest heteroatom from the molecular center of mass (COM), farthest atom from the molecular COM, number of heteroatoms, molecular surface area (MSA), and theoretically computed CCS (label). The COM and MSA were calculated using ASE^30^ (Atomic Simulation Environment) and the PyMOL^31^ API, respectively.

### ML Framework and Neural Network Architecture

The ML model was built using the TensorFlow^32,33^ open-source platform. Within TensorFlow we implemented the Keras^34^ deep learning API Sequential model as the deep neural network (DNN) architecture for the machine learning process. The Sequential model is appropriate for handling data with a linear topology, which we presumed to exist between feature(s) and label, and for computational efficiency. The DNN architecture has an input layer, six hidden layers, and an output layer. The initial input layer consists of four nodes (each containing a single feature representation), six hidden layers of various node counts, and an output layer with a single node for the label. Moreover, each hidden layer is activated by either the “relu” or “gelu” activation function.^35^ To handle prediction performance, the mean absolute error, “accuracy”, and “Adam” are selected as the loss function, the metrics, and the optimizer, respectively (for additional hyperparameter settings, see Table S1 in the Supporting Information). The training and validation involved an 80/20 split, respectively, of the total data set. See Figure S2 for the model CCS prediction error for a validation set of 242 conformations. The test set was completely outside from the original data set. This helps to prevent training data leakage and ensure that the performance on the test set is genuine.

### Software Specifications

To make the ML model executable with the intended CCS knowledge-based focusing operation, we developed a Python program platform using the Google Colab notebook, with this specification written explicitly into the program. The program takes in an input XYZ formatted file and generates test data from which the five features are extracted and submitted into the input layer of the Keras DNN for CCS prediction. The relevant CCS filter size is automatically set in response to the user input CCS value and the prediction performance (accuracy) of the model at its latest training update (retraining and/or a new data update will change the accuracy). Moreover, the upper limit and lower limit of the CCS filter are determined semiautonomously by the program using eqs 1–4, respectively.
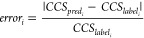
1
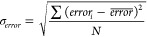
2
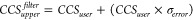
3
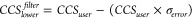
4Here, *CCS*_*pred*_ is the model predicted (or hypothesis) CCS, *CCS*_*label*_ is the training data label, and *CCS*_*user*_ is the user input CCS value (*e.g*., experimental reference). *error* is the average batch error, *error*_*i*_ is the individual prediction error, *N* is the total number of predictions, and *σ*_*error*_ is the standard error. The conformers that fall within the CCS filter range (*i.e*., upper limit and lower limit) are allocated and saved in a new folder for further downstream processing. In a scenario where the conformational space sampled is large, there is an option to set a capacity limit for the number of relevant conformers to be saved in case the filter captures relevant conformers in excess. The data set and executable program for CCSF is available at https://github.com/mitkeng/CCS_Focusing.

## Results and Discussion

### Lipid Structure Prediction Performance and Training Data Procurement using the Standard Workflow

We ran the standard workflow on 20 lipid^19−23^ species of various degrees of unsaturation to gauge the effect of structural flexibility on structure prediction performance. The results in Table 1 show that on average, the gas phase equilibrium lipid structures that we have predicted are not far off the 3% error threshold. We noticed, however, that the more saturated fatty acids (*e.g*., vaccenic acid, petroselinic acid, and elaidic acid) have a higher frequency of suboptimal structure prediction, obtaining a CCS error of greater than the acceptable 3% individually. Most notable is the *trans* vaccenic acid, which has a dramatic CCS error of ∼35%. Our preliminary result for the *cis* vaccenic acid shows that it is ∼7 kcal/mol less stable in the gas phase, which assures us that the reference CCS value has been correctly assigned to the *trans* configurational isomer. Interestingly, vaccenic, oleic, petroselinic, and elaidic acids are isomers that differ only in the position of their one double bond. This subtle distinction is apparently enough to compromise the conformer sampling process. For now, we can reasonably speculate that the large error for vaccenic acid may be a consequence of either experimental error (*e.g.,* analyte contamination, signal misidentification, dimerization, clustering/declustering, or instrument error) or a deficiency in our current computational approach to sampling relevant conformers. It should be remarked that the large error increases the standard deviation (±7%) and makes the transferability of our current method an issue. To delineate the root cause of this error, we reran vaccenic acid, along with eight other lipid systems (designated in Table 1), through the ML augmented workflow that implements the CCS knowledge-based focusing.

**Table 1:** Gas-phase structure prediction results were obtained using the standard workflow^c^

	Lipid [M-H]-	Carbon Chain	π-Bond Position	Exp. CCS (Å^2^)	Cal. CCS (Å^2^)	% Error	ref.
1	Vaccenic acid^a^	18	tranΔ^11^	174.95	235.72	34.74	(21)
2	Oleic acid^a^	18	cisΔ^9^	175.22 ± 1	177.16	1.11	(19,20,22)
3	Petroselinic acid^a^	18	cisΔ^6^	175.50	183.34	4.47	(23)
4	Elaidic acid^a^	18	tranΔ^6^	175.90	188.99	7.44	(24)
5	α-Linolenic acid	18	Δ^9,12,15^	174.26 ± 1	168.99	3.02	(19,20)
6	γ-Linolenic acid^a^	18	Δ^6,9,12^	174.77	181.54	3.87	(23)
7	Dihomo-γ-linolenic acid	20	Δ^8,11,14^	182.36 ± 1	184.54	1.20	(19,20)
8	Eicosatrienoic acid^a^	20	Δ^11,14,17^	178.70^b^	178.13	0.32	(26)
9	5(S)-HETE^a^	20	Δ^6,8,11,14^	186.27	177.50	4.71	(19)
10	12(R)-HETE	20	Δ^5,8,10,14^	183.18	184.93	0.96	(19)
11	15(S)-HETE	20	Δ^5,8,11,13^	184.77	190.10	2.88	(25)
12	15-OxoETE^a^	20	Δ^5,8,11,13^	183.13	195.25	6.62	(25)
13	14(15)-EpETE^a^	20	Δ^5,8,11,17^	183.90	177.20	3.64	(25)
14	5-OxoETE	20	Δ^6,8,11,14^	185.13	180.58	2.46	(25)
15	15(S)-HEPE	20	Δ^5,8,11,13,17^	183.07	189.06	3.27	(25)
16	7(R)-Maresin-1	22	Δ^4,8,10,12,16,19^	192.57	199.05	3.37	(19)
17	8(S)-HETE	20	Δ^5,9,11,14^	184.14	188.89	2.58	(19)
18	7(S)-Maresin-1	22	Δ^4,8,10,12,16,19^	192.54	199.75	3.74	(19)
19	PGE1	20	Δ^9,11,15^	190.23	192.52	1.20	(25)
20	PGF2α	20	Δ^9,11,15^	192.34 ± 2	190.13	1.15	(19,25)
	Average					4.64 ± 7	

a Systems selected for validation set.

b Reference CCS value obtained using the CCSbase CCS prediction webserver.

c The candidate conformation for each lipid system was selected by scoring the DFT (D3BJ-B3LYP/6-31+G(d,p)) relative energy. The % error is the difference between the experimental and calculated CCS. The Δ represents the carbon number where the double bond is located.

Before we move on to explore the performance of the augmented method, we first want to elaborate more on the training data set since we only briefly mentioned it earlier. Moreover, for the data set we exclusively used the successfully computed CCS results from the standard workflow and the corresponding raw conformers of the 20 lipid systems tabulated in Table 1 to train the model. These lipid ions range in size from 18 carbon chains to 22 carbon chains of the mono- and polyunsaturated types. We believe that this data set should provide enough topological and chemical diversity within the lipid class for the model to be effective despite the small number of unique systems sampled.

### Performance Validation of Model on Structure Prediction Quality

**Figure 2:**
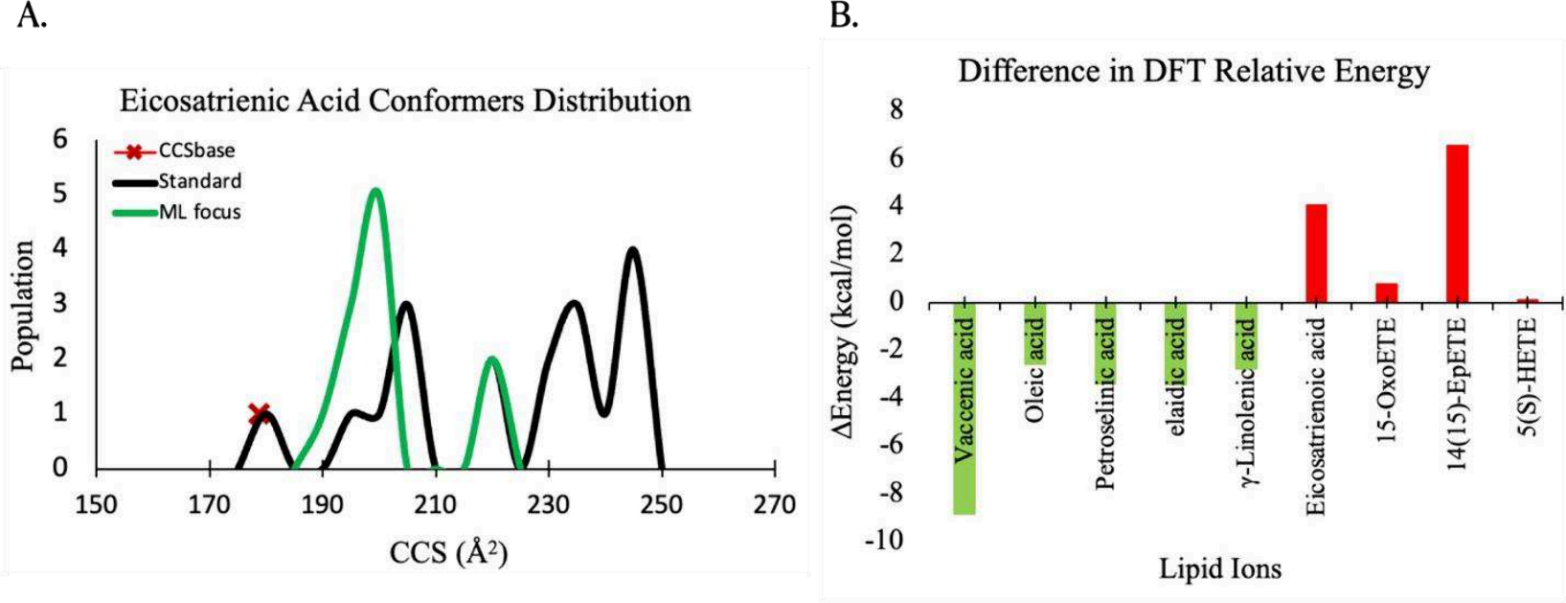
(A) The DFT optimized ensemble distribution for eicosatrienoic acid shows a failure by the model (green curve) to capture the experimental conformer (red cross) compared to that of the standard method without ML focusing (black curve). (B) The difference in DFT (D3BJ-B3LYP/6-31+G(d,p)) energies for the equilibrium candidate conformations generated using the standard method versus the ML augmented method. Green bar signifies that the minimum energy structure is obtained using the augmented method, and the red bar the minimum is found by the standard method.

For the ML augmented workflow, an initial ensemble of 11000 conformers was generated for each of the nine rerun lipid systems, which equates to more than ten times the number of conformers employed for the standard workflow. After CCSF and Autograph clustering there are typically less than 50 representative conformers remaining for the rerun for the remaining downstream steps. In general, we found that the number of representative conformers per system going into the QM step for the augmented workflow is similar to the standard workflow; thus, we are able to maintain nearly the same computational expense at the bottleneck despite sampling a much larger computational space. Table 2 shows the results for the validation set structure predictions performed by using the ML augmented workflow. The average rerun CCS % error for the nine lipids improved to ∼2% from ∼7% for the same systems processed with the standard workflow. The favorable outcome unequivocally reinforces the benefit of sampling large conformational space when modeling flexible molecular systems. However, for eicosatrienoic acid, an eicosanoid, the structure predicted from the augmented method resulted in a much greater error (∼7.7% error) compared to the prediction made through the standard workflow (∼0.3% error). This result is surprising considering that the quality should have either remained the same or improved but not worsened after sampling more conformers. We see in Figure 2A that although CCSF produced a significantly narrower conformer spread, with an average ensemble CCS value of ∼199 ± 9 Å^2^, the larger conformational spread produced by the standard method fortuitously captured the eicosatrienoic acid conformer that best agreed with the reference value (*i.e*., 178.7 Å^2^) despite producing an average CCS of ∼220 ± 25 Å^2^ for the ensemble (for the distribution plots for the remaining validation ions, see Figure S3 in the Supporting Information). There are two likely possible sources for the decline in accuracy for eicosatrienoic acid with the augmented method: insufficient training examples or the inadvertent loss of experimentally relevant conformers in the clustering step due to pseudosimilarity in the structural topology from a highly concentrated conformational space.

**Table 2:** CCSF validation results using the ML augmented method^a^

	Lipid [M-H]-	Carbon Chain	π-Bond Position	Exp. CCS (Å^2^)	Cal. w/CCSF (Å^2^)	% Error
1	Vaccenic acid	18	tranΔ^11^	174.95	175.55	0.34
2	Oleic acid	18	cisΔ^9^	175.22 ± 1	171.77	1.97
3	Petroselinic acid	18	cisΔ^6^	175.50	171.78	2.12
4	Elaidic acid	18	tranΔ^6^	175.90	172.53	1.92
5	γ-Linolenic acid	18	Δ^6,9,12^	174.77	173.81	0.55
6	Eicosatrienoic acid	20	Δ^11,14,17^	178.70	192.41	7.67
7	5(S)-HETE	20	Δ^6,8,11,14^	186.27	184.77	0.81
8	15-OxoETE	20	Δ^5,8,11,13^	183.13	184.16	0.56
9	14(15)-EpETE	20	Δ^5,8,11,17^	183.90	187.64	2.03
	Average					2.00 ± 2

a The candidate conformation for each lipid system was selected by scoring the DFT (D3BJ-B3LYP/6-31+G(d,p)) relative energy. The % error is the difference between the experimental and calculated CCS.

The selected candidate structures for the validation set have been scored according to their respective ensemble DFT (*i.e*., D3BJ-B3LYP/6-31+G(d,p)) relative energies obtained using the ML augmented workflow; however, these structures may not necessarily represent the global gas phase equilibrium conformations when their electronic energies are compared across both the augmented and standard method. Figure 2B shows that the candidate structures produced for vaccenic, oleic, petroselinic, elaidic, and γ-linolenic acids through the augmented method are in fact the global minimum conformers in this work. On the other hand, the global minimum conformers for eicosatrienoic, 15-oxoETE, and 14(15)-EpETE are obtained using the standard workflow as evident by their lower DFT energies compared to the augmented method. For the lipid ion 5(S)-HETE, where we observe a very small ΔRE (relative energy) of ∼0.25 kcal mol^–1^, both Boltzmann-weighted equilibrium structures generated by the augmented and standard methods are found to be populated in the gas phase in a nearly 1 to 1 ratio. For the most part, our model successfully discovered new energy minima for the lipid systems within the validation set; however, we need assurance that the performance is authentic and not due to a selection bias of the training data set.

### Model Evaluation on Unseen Test Data

To carry out an unbiased performance evaluation of our knowledge-based CCSF model, we introduced a test data set consisting of unseen data extracted from ten new lipid systems of moderately diverse topology and chemical composition. It should be noted that we maintained, for now, the size of the test systems to the ≤22 carbon chain to support ML interoperability, since we have only trained our model on lipid ions having ≤22 carbon chains. The structure prediction results for the test systems following the augmented workflow protocol are shown in Table 3. Globally, the resolved structures using the augmented method continue to achieve good agreement with experiment, giving an average CCS error of <2%, which is desirable since it is within our CCS error tolerance for this work. Additionally, we carried out a feature similarity survey to assess whether the favorable performance here is due to good model generalizability or a data similarity effect. This is done by taking the % difference of three features, *i.e*., farthest heteroatom from COM (Feature 1), farthest atom from COM (Feature 2), and MSA (Feature 3), between the training set and the test set using
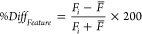
where *F*_*i*_ is a feature from the training set. *F̅* is obtained by taking the average values for Feature 1, Feature 2, or Feature 3 of the candidate conformers (ensemble) for each lipid system in the test set. The results are tabulated in Table 4 as averages for simplicity (for more detail, see Figure S4 in Supporting Information). Globally, the values of the features for the test set are significantly different from the training data set, and, thus, we can conclude that for this batch of unseen test data the overall performance can be attributed to good model generalizability.

**Table 3:** Performance results of the ML model on unseen test dataset^a^

	Lipid [M-H]-	Carbon Chain	π-Bond Position	Exp. CCS (Å^2^)	Cal. w/CCSF (Å^2^)	% Error	ref.
1	Adrenic acid	22	Δ^7,10,13,16^	189.97	185.26	2.48	(19)
2	DHA	22	Δ^4,7,10,13,16,19^	190.17	189.75	0.22	(19)
3	Carb. Thromb. A2	22		201.10	197.87	1.61	(19)
4	Artemisic acid	18	Δ^9,11^	182.20	189.44	3.97	(19)
5	14S-HDHA	22	Δ^4,7,10,12,16,19^	189.29	180.43	4.68	(19)
6	13-Oxo-ODE	18	Δ^9,11^	179.53	175.42	2.29	(19)
7	17S-HDHA	22	Δ^4,7,10,13,15,19^	193.67	191.94	0.89	(19)
8	Gondoic acid	20	cisΔ^11^	183.69 ± 1	187.64	2.15	(19,20)
9	Glutarylcarnitine	5		165.30	167.25	1.18	(23)
10	Lipoxin A4	20	Δ^7,9,11,13^	193.03	192.01	0.53	(19)
	Average					2.0 ± 1.5	

a The candidate conformation for each lipid ion was selected by scoring the DFT (D3BJ-B3LYP/6-31+G(d,p)) relative energy. The % error is the difference between the experiment and calculated CCS. Note: considering the carbon chain size and unsaturation, the ions listed here are still within the limit of interpolation.

**Table 4:** Averages of the percent difference between features of the training set and test set^a^

Lipid Ions	Feature 1	Feature 2	Feature 3
Adrenic acid	13.71	25.00	–2.67
DHA	9.07	15.51	–1.66
Carb. Thromb. A2	7.41	10.08	–5.78
Artemisic acid	23.41	22.59	3.69
14S-HDHA	20.27	24.56	–3.25
13-Oxo-ODE	25.11	23.48	7.44
17S-HDHA	8.99	19.18	–4.03
Gondoic acid	19.20	21.82	–4.45
Glutarylcarnitine	33.90	49.95	32.31
Lipoxin A4	1.14	15.24	–6.33

a The training set contains 716 data points (instances). The features for the lipid systems in the test set are averages of the conformers in the ensemble. Values are in percent.

When we look at the model’s performance on a system-to-system basis, we observe that only artemisic acid and 14S-HDHA have unacceptable CCS errors, which in consequence gives our model an 80% reproducible structure prediction success rate. Moreover, it is worth mentioning that there is nothing obviously unfamiliar about the chemical composition of artemisic acid or 14S-HDHA that would justify the suboptimal accuracy observed. For example, 14S-HDHA and 17S-HDHA are isomers that differ in the position of a single hydroxyl group at the hydrocarbon tail, yet the former has an ∼4.7% CCS error, while the latter has a <1% error. With this in mind, we hypothesize that the disparity in the error between the two isomers is likely attributed to the deficiency of experimentally relevant conformers within the ensemble of 11000 conformers for 14S-HDHA. This hypothesis can be potentially tested by increasing ConfGen output to >11000 conformers.

Thus far, we have operated the model on test systems that are within the training set molecular size limit of 22 carbon chains for exclusively unsaturated lipid species. Here, we evaluate the model’s performance at extrapolating CCS values for lipids with carbon chain ≥22. The lipids chosen for this task are γ-tocopherol, lignoceric acid, nervonic acid, and tricosylic acid, which range in size from 22 C-chain to 24 C-chain, with three out of the four having fully saturated hydrocarbon tails. As we have already noted, the lipids used in the training set have ≤22 carbon chain and some degree of unsaturation, and thus, these unfamiliar features may pose a significant challenge for our model, which is often the case with any ML prediction based on extrapolation outside of its training space. When CCSF was executed on the ensembles (*i.e.,* 11000 conformers) for the four new lipid ions, no experimentally viable gas phase structures were captured by the program. Although this outcome is not entirely unexpected, it is a surprise, however, considering that the minimum CCS forecasted by the ML model for each lipid ensemble overestimates the experimental CCS (Table 5), with an average CCS error of ∼29% and the largest error being ∼41% (lignoceric acid). Notably, these errors are much larger than the CCS filter size set by our program. It is possible that the model performance degraded sharply as we moved out of the range of interpolation established by the training. Our hypothesis is valid considering that lipids in the training set have on average 14 rotatable bonds (mode = 14), whereas the test set in Table 5 has an average of 19 rotatable bonds (mode = 21); on the contrary, the average rotatable bonds for the test set in Table 3 is ∼13.7 (mode = 14). This reasonably explains the difference in performances between the two test sets. Thus, more rotatable bonds translates to an estimated 3^N^ (where N is the number of rotatable bonds) exponential increase in conformational space that the model must account for.^36^ Consequently, since there are no relevant conformers captured through ML CCSF, we were unable to engage the augmented workflow and, therefore, chose instead to carry out only the standard workflow on the four lipids using ensembles of 1000 conformers.

**Table 5:** Results for ML model extrapolation study on unseen data^a^

	Lipid [M-H]-	Carbon Chain	π-Bond Position	Exp. CCS (Å^2^)	Cal. w/CCSF (Å^2^)	% Error	ref.
1	γ-tocopherol	22		207.14	241.98	16.82	(19)
2	Lignoceric acid	24		200.36 ± 1	283.11	41.30	(19,20)
3	Nervonic acid	24	Δ^15^	197.48	245.13	24.13	(23)
4	Tricosylic acid	23		196.88	263.54	33.86	(23)
	Average					29.03 ± 11	

a The lipid ions listed contain unfamiliar size (larger C chain size) and saturation not found in the training dataset. The minimum predicted CCS is the smallest CCS value predicted (without DFT) per lipid ion raw ensemble. The % error is the difference between the experiment and the model predicted CCS.

**Figure 3:**
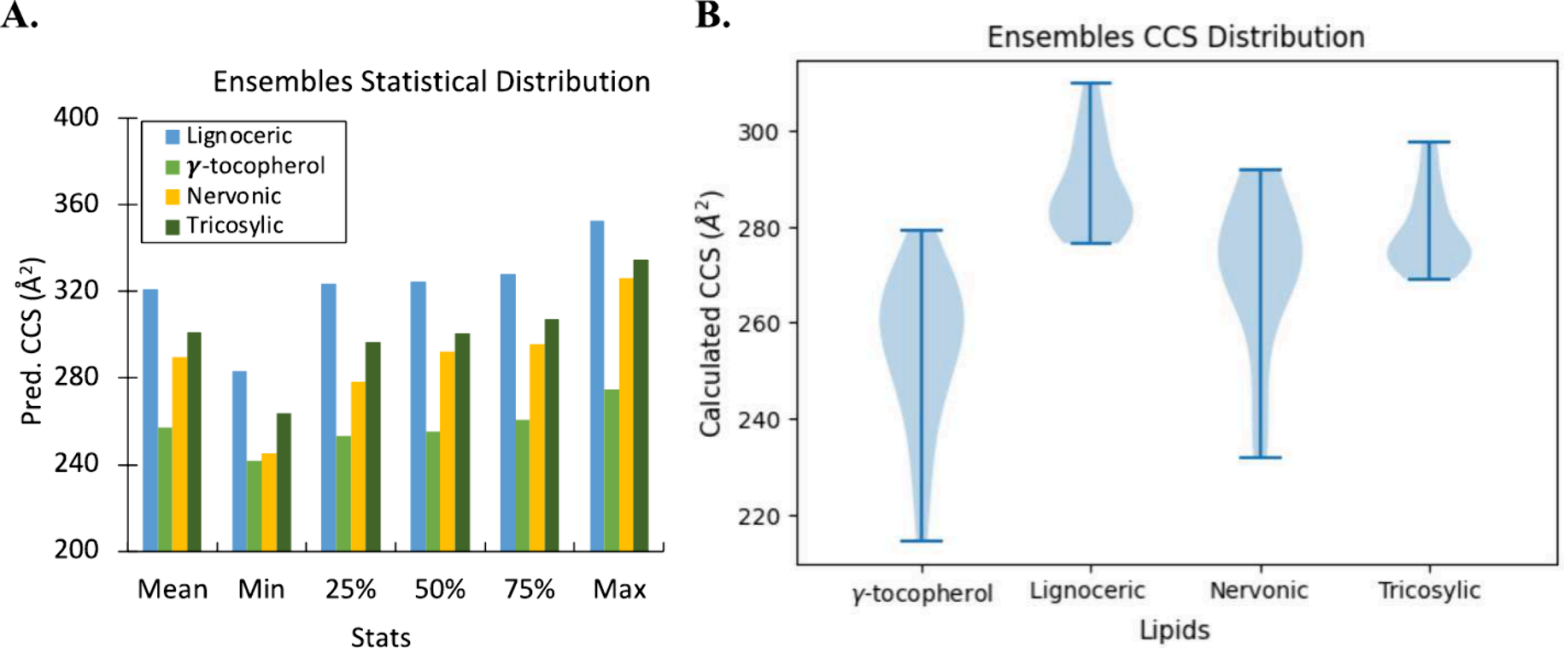
Ensembles of conformer distributions for the (A) CCS predicted (upstream) by the model and (B) the CCS computed for the DFT (D3BJ-B3LYP/6-31+G(d,p)) optimized conformers using the standard method without ML focusing.

**Figure 4:**
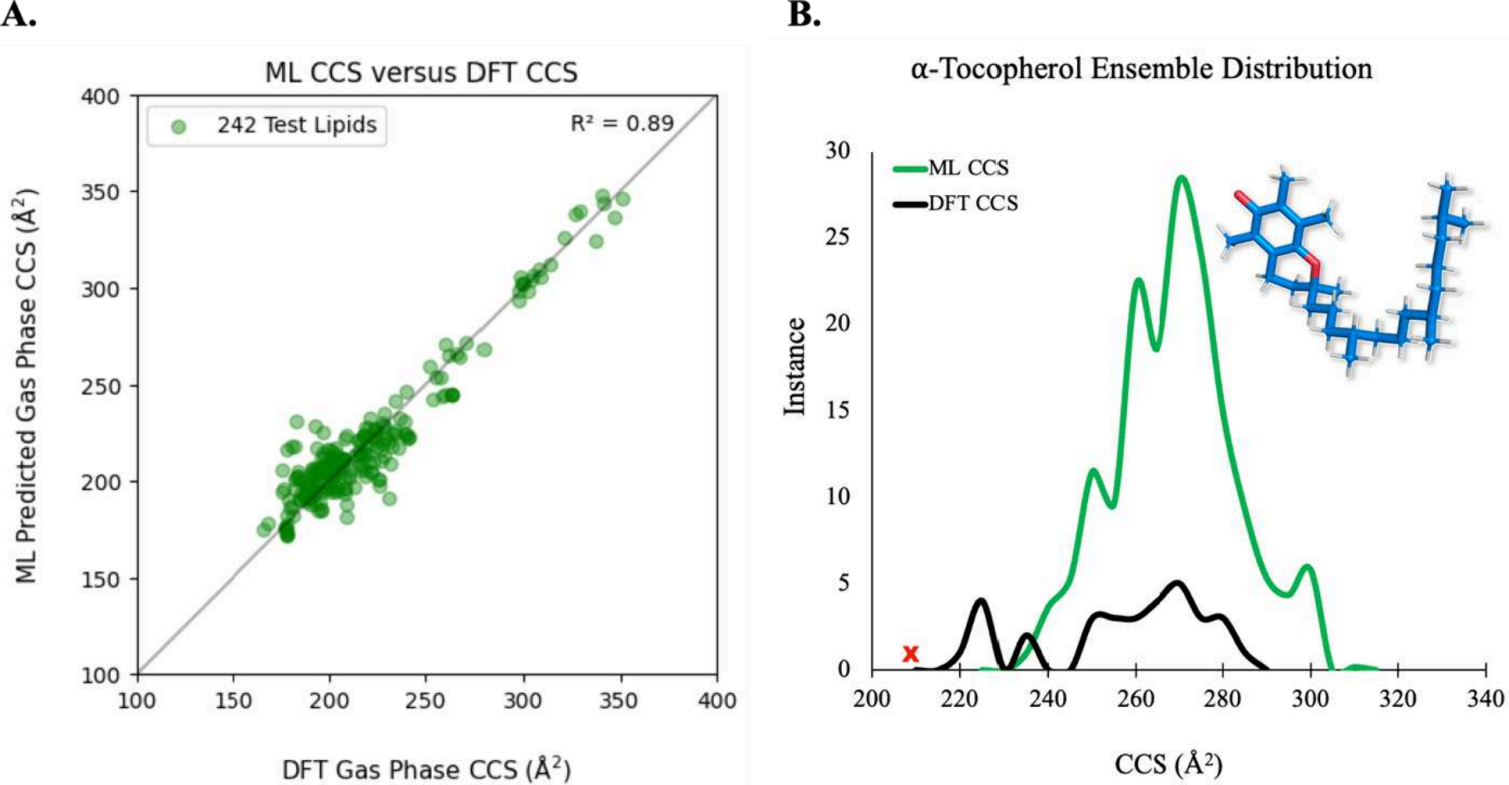
(A) Final model performance at predicting DFT derived CCS values for 242 test systems. The test set is 20% of ∼1200 total in data set for this run. (B) Distributions of CCS predicted for α-tocopherol (blue structure) raw conformers by ML model (green) and CCS computed from DFT equilibrium conformations (black). Experimental CCS value is denoted by the red x.

It is necessary that we obtain the CCS for the DFT optimized structures, because this helps to determine whether the ML model has poor generalizability or whether the experimental disagreement observed is a systemic issue related to modeling large, flexible molecules. The preliminary results hereafter for the standard method reveal that the CCS distributions for the DFT optimized ensembles (Figure 3A,B) are similar to the CCS distributions generated by the ML model except for γ-tocopherol. Likewise, the results for the completed standard workflow (Table 6) show high CCS errors (average error of ∼26%) for the final resolved candidate conformations that are comparable to those the model predicted for the minimum CCS values, but again, except for γ-tocopherol. The CCS errors for γ-tocopherol are ∼3.5% and ∼17% for the standard method and for the minimum CCS that the model predicted, respectively. We hypothesize that the error discrepancy between the standard and augmented method may have resulted from the lack of training exposure to a γ-tocopherol-type chemical structure (*i.e*., fused benzene and oxane rings with a long flexible hydrocarbon tail). In response, we added γ-tocopherol along with the remaining lipid systems from Table 1 and Table 3 to the data set and retried CCSF on α-tocopherol. Figure 4A shows the latest prediction performance (*i.e*., R^2^ = 0.89) from the increase in the training instances, and Figure 4B illustrates the distributions for the model-predicted CCS versus the DFT-derived CCS values for α-tocopherol. Despite the additional training and good overlap in CCS distributions, the model did not pick up any relevant conformer within the experimental range set from an ensemble of 10000 raw conformers. Moreover, the equilibrium structure for α-tocopherol obtained by DFT (*i.e*., D3BJ-B3LYP/6-31+G(d,p)) has a suboptimal CCS error of ∼4.6% (218.85 Å^2^), whereas the lowest CCS value predicted by the model is ∼233 Å^2^. Despite the additional effort, the tocopherol lipid type still poses a challenge for our model and thus will require more input training examples in the future as we receive more data.

**Table 6:** Structure prediction results using the standard method^a^

	Lipid [M-H]-	Carbon Chain	π-Bond Position	Exp. CCS (Å^2^)	Cal. w/CCSF (Å^2^)	% Error
1	γ-tocopherol	22		207.14	214.47	3.54
2	Lignoceric acid	24		200.36 ± 1	288.92	44.20
3	Nervonic acid	24	Δ^15^	197.48	232.92	17.95
4	Tricosylic acid	23		196.88	276.03	40.20
	Average					26.44 ± 19

a The candidate conformation for each lipid ion was selected by scoring the DFT (D3BJ-B3LYP/6-31+G(d,p)) relative energy. The % error is the difference between the experiment and calculated CCS.

Nonetheless, the failure to accurately obtain good experimental agreement for some systems using the standard method provides an excellent example of how important it is to forecast results early on in a modeling workflow to prevent wasted effort and time on nonviable ensembles. Simply put, our model had already predicted prior that the ensembles generated for the four test lipid ions (*i.e*., Table 5) have very poor agreement with experiment, and, in retrospect, we could have preemptively opted not to proceed directly to the expensive QM step and instead generate more conformers or use a different conformation generator entirely. Altogether, the latest results indicate that the model is fairly generalizable and that the large errors that we have encountered are likely a consequence of the inefficient production of relevant conformers at the conformational generation step.

## Conclusion

We first presented in this work the method and process used in the development of a novel CCS knowledge-based machine learning model for finding relevant gas phase conformations within a raw ensemble for fatty acids with 22 or less carbon atoms. Furthermore, by restricting the input features in the DNN infrastructure to relevant molecular descriptors that correlate well with the CCS label, we managed to achieve good prediction performance with minimal adverse impact on the overall computational expense. It should be remarked that, though currently only 34 unique systems have been used to train our model, the features in combination with each other are unique and offer distinct data points (>1100) for model training. Furthermore, the infusion of new data and subsequent training updates will be ongoing as we continue to further process more unique lipid systems.

We have integrated the ML model into an executable Python platform for a user-friendly interface that supports a seamless modeling workflow. Second, we reported successful results in both the validation study and in the test study that evaluated the model’s performance against unseen data. Additionally, the model also demonstrated good generalizability when challenged with extrapolating CCS for molecular species with unfamiliar size and flexibility but of a similar size range. We also show that extension to large fatty acids (which were outside the training set) does not perform as well using the ML augmented or the standard protocol. We show data that suggests this has to do with the conformational search step rather than the ML model, which supports that further development of conformational search tools tuned to handle large conformationally flexible lipids is still needed. This suggests that chains greater than the 22 carbon atoms remain challenging to model. Altogether, we believe that the implementation of our CCS knowledge-based ML model into a modeling workflow is potentially beneficial for both results quality and results turnaround time.

## Data Availability

The XYZ files for the lipid systems and their tabulated DFT results, ML data set, trained model file, and link to the CCS Focusing Google Colab notebook are accessible at https://github.com/mitkeng/CCS_Focusing.

## Supporting Information Available

ML augmented workflow, hyperparameters setting, lipid test set CCS prediction errors, standard and augmented workflow distributions, and feature similarity analysis (PDF)

## References

[ref1] Cole J. C.; Korb O.; McCabe P.; Read M. G.; Taylor R. Knowledge-Based Conformer Generation Using the Cambridge Structural Database. J. Chem. Inf. Model. 2018, 58 (3), 615–629. 10.1021/acs.jcim.7b00697. 29425456

[ref2] Mendenhall J.; Brown B. P.; Kothiwale S.; Meiler J. BCL::Conf: Improved Open-Source Knowledge-Based Conformation Sampling Using the Crystallography Open Database. J. Chem. Inf. Model. 2021, 61 (1), 189–201. 10.1021/acs.jcim.0c01140. 33351632 PMC8130828

[ref3] Das S.; Merz K. M. Jr. Molecular Gas-Phase Conformational Ensembles. J. Chem. Inf. Model. 2024, 64 (3), 749–760. 10.1021/acs.jcim.3c01309. 38206321

[ref4] Hawkins P. C. D. Conformation Generation: The State of the Art. J. Chem. Inf. Model. 2017, 57 (8), 1747–1756. 10.1021/acs.jcim.7b00221. 28682617

[ref5] Beliakov G.; Lim K. F. Challenges of continuous global optimization in molecular structure prediction. European Journal of Operational Research 2007, 181 (3), 1198–1213. 10.1016/j.ejor.2005.08.033.

[ref6] Kaminský J.; Jensen F. Force Field Modeling of Amino Acid Conformational Energies. J. Chem. Theory Comput. 2007, 3 (5), 1774–1788. 10.1021/ct700082f. 26627621

[ref7] Kanu A. B.; Dwivedi P.; Tam M.; Matz L.; Hill H. H. Jr Ion mobility–mass spectrometry. Journal of mass spectrometry 2008, 43 (1), 1–22. 10.1002/jms.1383. 18200615

[ref8] Jurneczko E.; Barran P. E. How useful is ion mobility mass spectrometry for structural biology? The relationship between protein crystal structures and their collision cross sections in the gas phase. Analyst 2011, 136 (1), 20–28. 10.1039/C0AN00373E. 20820495

[ref9] Gabelica V.; Marklund E. Fundamentals of ion mobility spectrometry. Curr. Opin. Chem. Biol. 2018, 42, 51–59. 10.1016/j.cbpa.2017.10.022. 29154177

[ref10] Das S.; Tanemura K. A.; Dinpazhoh L.; Keng M.; Schumm C.; Leahy L.; Asef C. K.; Rainey M.; Edison A. S.; Fernández F. M.; Merz K. M. Jr. In Silico Collision Cross Section Calculations to Aid Metabolite Annotation. J. Am. Soc. Mass Spectrom. 2022, 33 (5), 750–759. 10.1021/jasms.1c00315. 35378036 PMC9277703

[ref11] Schrödinger Release 2023–3: Maestro; Schrödinger, LLC: New York, NY, 2023.

[ref12] Watts K. S.; Dalal P.; Murphy R. B.; Sherman W.; Friesner R. A.; Shelley J. C. ConfGen: A Conformational Search Method for Efficient Generation of Bioactive Conformers. J. Chem. Inf. Model. 2010, 50 (4), 534–546. 10.1021/ci100015j. 20373803

[ref13] Tanemura K. A.; Das S.; Merz K. M. Jr. AutoGraph: Autonomous Graph-Based Clustering of Small-Molecule Conformations. J. Chem. Inf. Model. 2021, 61 (4), 1647–1656. 10.1021/acs.jcim.0c01492. 33780248

[ref14] Gaussian 16, Rev. C.01; Gaussian, Inc.: Wallingford, CT, 2016.

[ref15] (acccessed 10/3/2023). Grimme S.; Antony J.; Ehrlich S.; Krieg H. A consistent and accurate ab initio parametrization of density functional dispersion correction (DFT-D) for the 94 elements H-Pu. J. Chem. Phys. 2010, 132 (15), 154104 10.1063/1.3382344. 20423165

[ref16] Grimme S.; Ehrlich S.; Goerigk L. Effect of the damping function in dispersion corrected density functional theory. J. Comput. Chem. 2011, 32 (7), 1456–1465. 10.1002/jcc.21759. 21370243

[ref17] Lukasz G. M.; Christopher J. G.; Sabine L. F.; Perdita E. B. A Careful Consideration of the Influence of Structure, Partial charges and Basis Sets on Collision Cross Sections of Monosaccharides when Comparing Values from DFT Calculated Conformers to those Obtained Experimentally. bioRxiv 2017, 162305 10.1101/162305.

[ref18] (acccessed 2023/02/16). Zanotto L.; Heerdt G.; Souza P. C. T.; Araujo G.; Skaf M. S. High performance collision cross section calculation—HPCCS. J. Comput. Chem. 2018, 39 (21), 1675–1681. 10.1002/jcc.25199. 29498071

[ref19] Zheng X.; Aly N. A.; Zhou Y.; Dupuis K. T.; Bilbao A.; Paurus V. L.; Orton D. J.; Wilson R.; Payne S. H.; Smith R. D.; Baker E. S. A structural examination and collision cross section database for over 500 metabolites and xenobiotics using drift tube ion mobility spectrometry. Chemical Science 2017, 8 (11), 7724–7736. 10.1039/C7SC03464D. 29568436 PMC5853271

[ref20] Stow S. M.; Causon T. J.; Zheng X.; Kurulugama R. T.; Mairinger T.; May J. C.; Rennie E. E.; Baker E. S.; Smith R. D.; McLean J. A.; Hann S.; Fjeldsted J. C. An Interlaboratory Evaluation of Drift Tube Ion Mobility–Mass Spectrometry Collision Cross Section Measurements. Anal. Chem. 2017, 89 (17), 9048–9055. 10.1021/acs.analchem.7b01729. 28763190 PMC5744684

[ref21] Zhou Z.; Shen X.; Tu J.; Zhu Z.-J. Large-Scale Prediction of Collision Cross-Section Values for Metabolites in Ion Mobility-Mass Spectrometry. Anal. Chem. 2016, 88 (22), 11084–11091. 10.1021/acs.analchem.6b03091. 27768289

[ref22] Hinz C.; Liggi S.; Mocciaro G.; Jung S.; Induruwa I.; Pereira M.; Bryant C. E.; Meckelmann S. W.; O’Donnell V. B.; Farndale R. W.; Fjeldsted J.; Griffin J. L. A Comprehensive UHPLC Ion Mobility Quadrupole Time-of-Flight Method for Profiling and Quantification of Eicosanoids, Other Oxylipins, and Fatty Acids. Anal. Chem. 2019, 91 (13), 8025–8035. 10.1021/acs.analchem.8b04615. 31074960 PMC7613057

[ref23] Nichols C. M.; Dodds J. N.; Rose B. S.; Picache J. A.; Morris C. B.; Codreanu S. G.; May J. C.; Sherrod S. D.; McLean J. A. Untargeted Molecular Discovery in Primary Metabolism: Collision Cross Section as a Molecular Descriptor in Ion Mobility-Mass Spectrometry. Anal. Chem. 2018, 90 (24), 14484–14492. 10.1021/acs.analchem.8b04322. 30449086 PMC6819070

[ref24] Picache J. A.; Rose B. S.; Balinski A.; Leaptrot K. L.; Sherrod S. D.; May J. C.; McLean J. A. Collision cross section compendium to annotate and predict multi-omic compound identities. Chemical Science 2019, 10 (4), 983–993. 10.1039/C8SC04396E. 30774892 PMC6349024

[ref25] da Silva K. M.; Wölk M.; Nepachalovich P.; Iturrospe E.; Covaci A.; van Nuijs A. L. N.; Fedorova M. Investigating the Potential of Drift Tube Ion Mobility for the Analysis of Oxidized Lipids. Anal. Chem. 2023, 95 (36), 13566–13574. 10.1021/acs.analchem.3c02213. 37646365

[ref26] Ross D. H.; Cho J. H.; Xu L. Breaking Down Structural Diversity for Comprehensive Prediction of Ion-Neutral Collision Cross Sections. Anal. Chem. 2020, 92 (6), 4548–4557. 10.1021/acs.analchem.9b05772. 32096630

[ref27] Causon T. J.; Hann S. Uncertainty Estimations for Collision Cross Section Determination via Uniform Field Drift Tube-Ion Mobility-Mass Spectrometry. J. Am. Soc. Mass Spectrom. 2020, 31 (10), 2102–2110. 10.1021/jasms.0c00233. 32812758

[ref28] Naylor C. N.; Schaefer C.; Zimmermann S. The dependence of reduced mobility, ion-neutral collisional cross sections, and alpha values on reduced electric field strengths in ion mobility. Analyst 2023, 148 (15), 3610–3621. 10.1039/D3AN00493G. 37404048

[ref29] Bisong E.. Google Colaboratory. In Building Machine Learning and Deep Learning Models on Google Cloud Platform: A Comprehensive Guide for Beginners, Bisong E., Ed.; Apress, 2019; pp 59–64.

[ref30] Hjorth Larsen A.; Jørgen Mortensen J.; Blomqvist J.; Castelli I. E; Christensen R.; Dułak M.; Friis J.; Groves M. N; Hammer B.ør.; Hargus C.; Hermes E. D; Jennings P. C; Bjerre Jensen P.; Kermode J.; Kitchin J. R; Leonhard Kolsbjerg E.; Kubal J.; Kaasbjerg K.; Lysgaard S.; Bergmann Maronsson J.; Maxson T.; Olsen T.; Pastewka L.; Peterson A.; Rostgaard C.; Schiøtz J.; Schutt O.; Strange M.; Thygesen K. S; Vegge T.; Vilhelmsen L.; Walter M.; Zeng Z.; Jacobsen K. W The atomic simulation environment—a Python library for working with atoms. J. Phys.: Condens. Matter 2017, 29 (27), 273002 10.1088/1361-648X/aa680e. 28323250

[ref31] The PyMOL Molecular Graphics System, Version 2.0; Schrödinger, LLC.

[ref32] Abadi M.; Barham P.; Chen J.; Chen Z.; Davis A.; Dean J.; Devin M.; Ghemawat S.; Irving G.; Isard M.; Kudlur M.; Levenberg J.; Monga R.; Moore S.; Murray D. G.; Steiner B.; Tucker P.; Vasudevan V.; Warden P.; Wicke M.; Yu Y.; Zheng X. TensorFlow: a system for large-scale machine learning. In OSDI’16: Proceedings of the 12th USENIX conference on Operating Systems Design and Implementation, Savannah, GA, USA, Nov 2, 2016; USENIX Association, 2016.

[ref33] Abadi M.; Agarwal A.; Barham P.; Brevdo E.; Chen Z.; Citro C.; Corrado G. S.; Davis A.; Dean J.; Devin M.; Ghemawat S.; Goodfellow I.; Harp A.; Irving G.; Isard M.; Jia Y.; Jozefowicz R.; Kaiser L.; Kudlur M.; Levenberg J.; Mane D.; Monga R.; Moore S.; Murray D.; Olah C.; Schuster M.; Shlens J.; Steiner B.; Sutskever I.; Talwar K.; Tucker P.; Vanhoucke V.; Vasudevan V.; Viegas F.; Vinyals O.; Warden P.; Wattenberg M.; Wicke M.; Yu Y.; Zhen X. TensorFlow: Large-Scale Machine Learning on Heterogeneous Distributed Systems. arXiv 2016, 10.48550/arXiv.1603.04467.

[ref34] Chollet F.; others. Keras: The Python Deep Learning library. Astrophysics Source Code Library 2018, ascl:1806.1022.

[ref35] Dubey S. R.; Singh S. K.; Chaudhuri B. B. Activation functions in deep learning: A comprehensive survey and benchmark. Neurocomputing 2022, 503, 92–108. 10.1016/j.neucom.2022.06.111.

[ref36] Pracht P.; Grimme S.; Bannwarth C.; Bohle F.; Ehlert S.; Feldmann G.; Gorges J.; Müller M.; Neudecker T.; Plett C.; Spicher S.; Steinbach P.; Wesolowski P. A.; Zeller F. CREST—A program for the exploration of low-energy molecular chemical space. J. Chem. Phys. 2024, 160 (11), 0197592 10.1063/5.0197592. 38511658

