# Supplementary material for: Eliminating the Deadwood: A Machine Learning Model for CCS Knowledge-Based Conformational Focusing for Lipids
Source: J Chem Inf Model. 2024 Oct 8;64(20):7864–72. doi: 10.1021/acs.jcim.4c01051 (PMC11523073; doi:10.1021/acs.jcim.4c01051)

Eliminating the Deadwood: A Machine Learning Model for CCS Knowledge-Based Conformational Focusing for Lipids

Mithony Keng and Kenneth M Merz, Jr.*

Department of Chemistry, Michigan State University,

East Lansing, Michigan 48824, United States

Department of Biochemistry and Molecular Biology, Michigan State University,

East Lansing, Michigan 48824, United States

*Corresponding Author: Kenneth M. Merz

*Corresponding Author Email: merz@chemistry.msu.edu

Supporting Information

Figure S1. A schematic of the CCS knowledge-based ML augmented workflow for structure prediction.

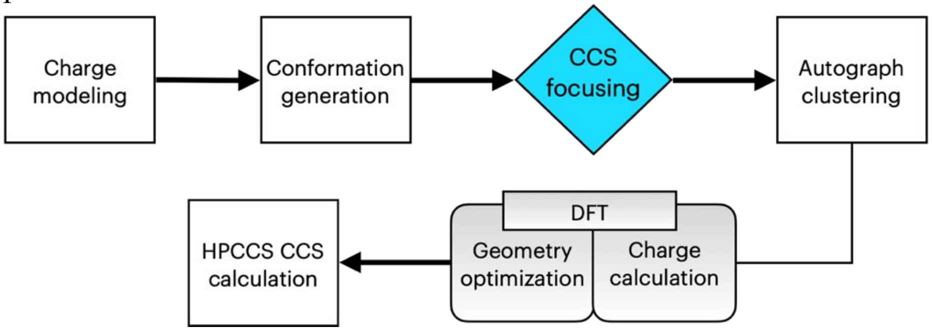

Table S1. Hyperparameters setting in python format used in training the CCS knowledge-based ML model for this work.

**Hyperparameters**	**Setting**
Training split	0.8
Validation split	0.2
Activation function	relu, gelu
Loss function	mean absolute error
Metrics	accuracy
Optimizer	Adam
Learning rate	0.001
Epoch	400

Figure S2. ML model CCS prediction error distribution for 242 validation lipid conformations.

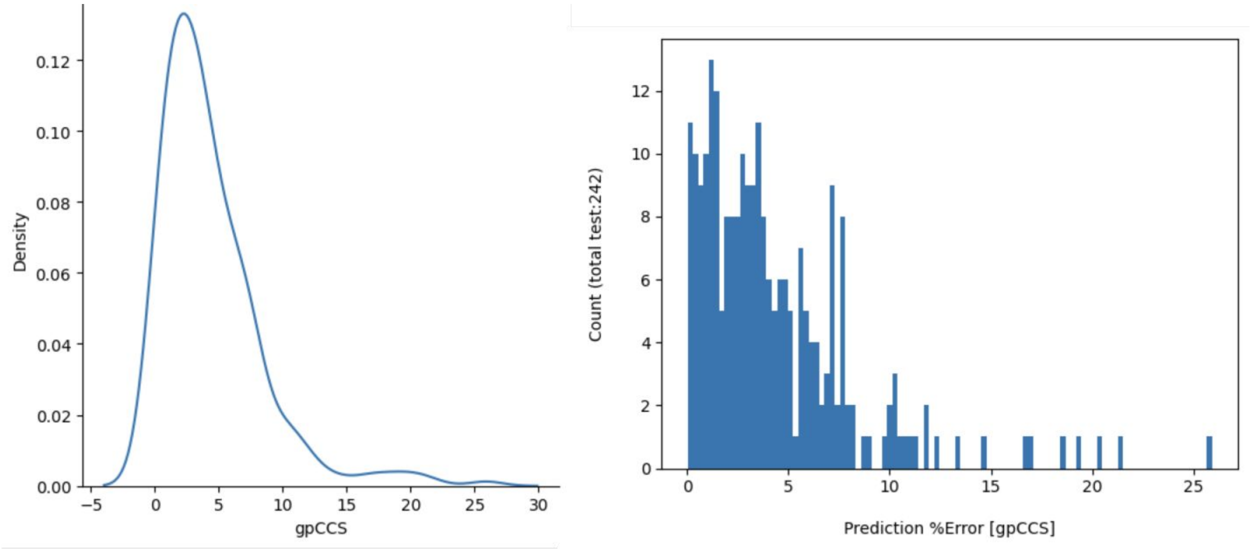

Figure S3. The DFT optimized ensemble distributions for the validation set ions A) vaccenic, B) oleic, C) petroselinic, D) elaidic, F) γ-linolenic, E) 5(S)-HETE, G) 15-OxoETE, and H) 14(15)-EpETE using the ML augmented method (green) and the standard method (black). The experimental reference CCS is indicated by the red cross.

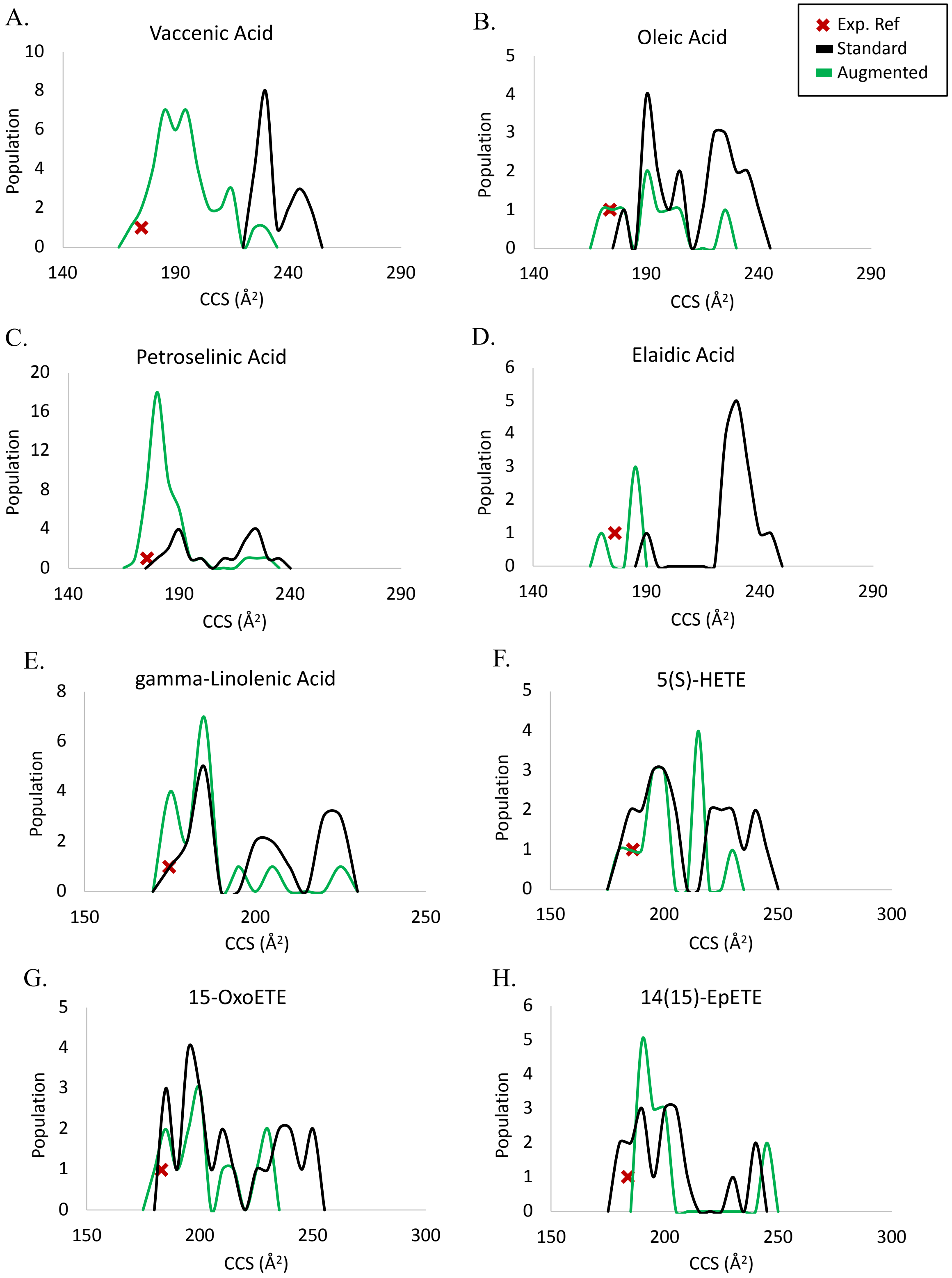

Figure S4. The % difference of three features, farthest heteroatom from COM (Feature 1; red), farthest atom from COM (Feature 2; green), and surface area (Feature 3; blue) between the training set and the test set. The test set, consisting of 10 lipid systems (names are above the graphs) and their averaged features, are contrast against 716 data points of corresponding training set features.

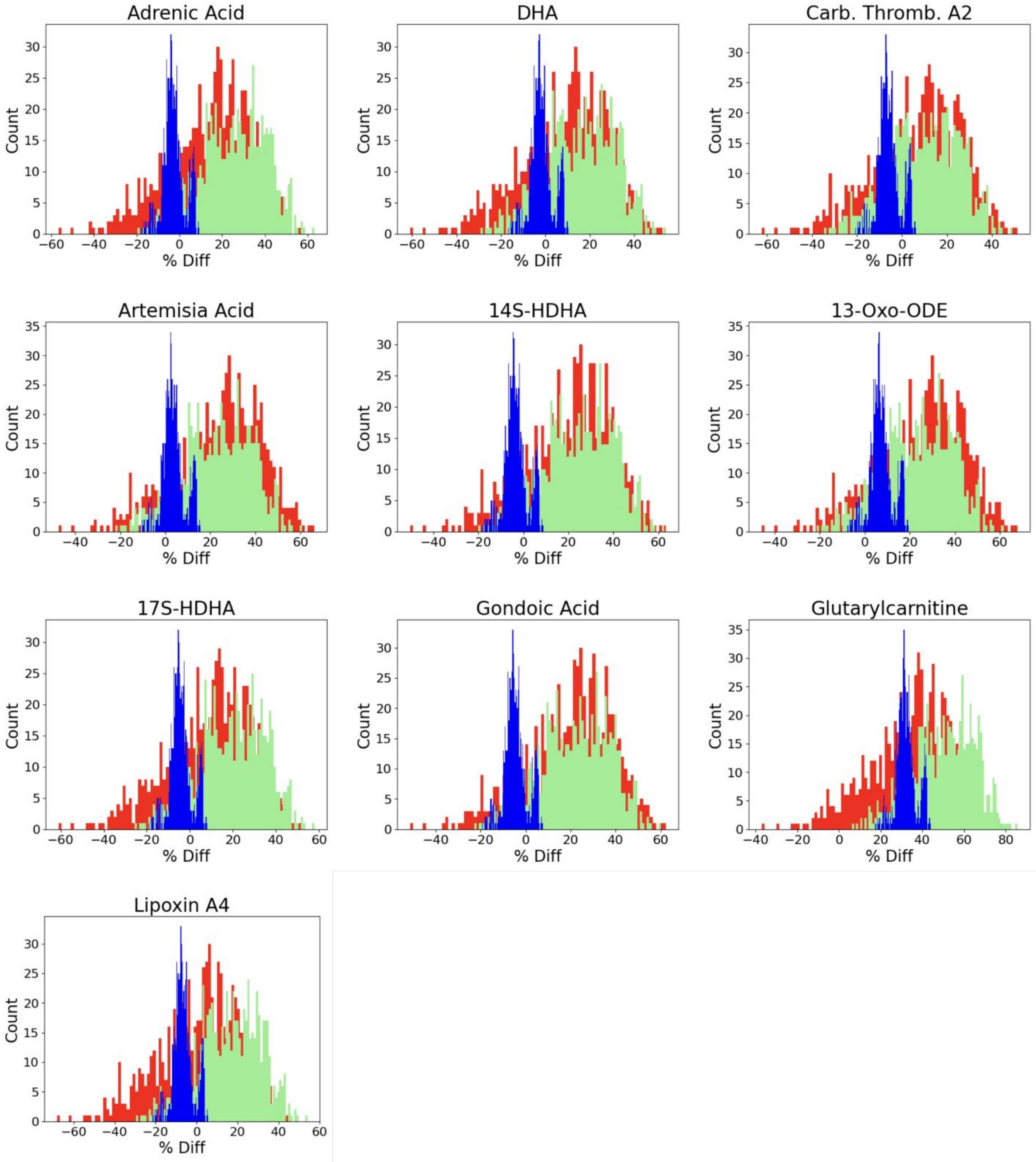

Supplement: Supplementary file 1 — ci4c01051_si_001.pdf [file ci4c01051_si_001.pdf]
